# Trogocytosis at the crossroad of cancer and immunity: mechanisms, implications and therapeutic perspectives

**DOI:** 10.3389/fcell.2025.1676945

**Published:** 2025-10-17

**Authors:** Aishwarya Guha, Saptak Banerjee

**Affiliations:** Department of Immunoregulation and Immunodiagnostics, Chittaranjan National Cancer Institute, Kolkata, India

**Keywords:** pro-tumorigenic trogocytosis, anti-tumorigenic trogocytosis, tumor microenvironment, chemoresistance, metastasis, CAR-immune cell fraticide

## Abstract

Trogocytosis, a rapid and contact-dependent exchange of plasma membrane fragments and associated molecules between cells, has recently emerged as a critical but underappreciated player in cancer biology. Traditionally studied in the context of immune cell communication, trogocytosis is now recognized for its paradoxical role in modulating tumor progression and therapeutic response across a broad spectrum of malignancies. This review highlights the novel and dynamic functions of trogocytosis in shaping the tumor microenvironment (TME), promoting immune evasion and influencing metastatic potential. Notably, cancer cells exploit trogocytosis to acquire immune regulatory molecules such as CD45, CD4 and checkpoint proteins, effectively dampening anti-tumor responses while enhancing their own survival. Simultaneously, immune effector cells including macrophages, T cells and natural killer (NK) cells leverage trogocytosis to recognize, attack and even kill tumor cells through mechanisms such as trogoptosis. Compelling new evidence also links trogocytosis to therapeutic resistance, particularly in chimeric antigen receptor (CAR-T and CAR-NK) cell therapies, where tumor antigens like CD19 and CD22 are siphoned off by effector cells, leading to T cell fratricide, functional exhaustion and tumor relapse. Beyond its biological significance, trogocytosis is gaining attention as a translational tool in oncology. It offers a novel platform for antigen-specific drug delivery, spatially restricted immune modulation and biomarker discovery through the detection of trogocytosed molecules on circulating immune-cells or extracellular vesicles. These findings redefine trogocytosis as not merely a passive membrane exchange process, but a central mechanism of intercellular communication with profound implications for cancer progression, immunotherapy and precision medicine.

## 1 Introduction

The notion of trogocytosis was initially introduced in the early 1970s, following the discovery of B-cell-derived immunoglobulins on the surface of mice thymus and activated lymphocytes. (Kim et al., 2025). During this process of trogocytosis, the lymphocytes actively receive portions of the plasma membrane fragments from the presenting cell during cell-cell interactions (Kim et al., 2025; Joly and Hudrisier, 2003). In-contrast to phagocytosis, which involves engulfment and lysis of the target cell via phagolysosome formation, trogocytosis involves transfer of membrane and cytosolic components from the target cell. This ensures substantial loss of functional membrane properties from the target cells, eventually leading to their death via trogoptosis, a form of trogocytosis intervened apoptosis (Kim et al., 2025). Moreover, trogocytosis is not restricted to lymphocytes but is actively engaged in macrophages, dendritic cells, natural killer (NK) cells and basophils mediated immune regulation. Apart from the immune-cells, trogocytosis is also functional in numerous non-immune-cells like the stromal cells and most importantly, it mediates the process of immune-evasion by the tumor cells (Kim et al., 2025; Zeng and Schwarz, 2020).

Miyake and Karasuyama, 2021 reported that receptor-ligand interactions, including Fcγ receptor and TCR-MHC (T cell receptor - major histocompatibility complex) binding, are central to initiating trogocytosis. MHC comprises of a set of cell surface glycoproteins that play fundamental role in antigen presentation and immune recognition. MHCs are broadly of two types MHC class I and MHC class II. MHC class I molecules are expressed on almost all nucleated cells and present endogenously derived peptides to CD8^+^ cytotoxic T lymphocytes, whereas MHC class II molecules are primarily expressed on antigen-presenting cells (APCs) such as dendritic cells, macrophages and B cells. They present exogenous peptides to CD4^+^ helper T lymphocytes. Through this antigen presentation, MHC molecules enable T-cell activation, immune-surveillance and maintenance of self and non-self discrimination. During trogocytosis, MHC molecules can be transferred from APCs to neighbouring immune or non-immune cells through this process, effectively converting them into non-professional APCs. In the tumor microenvironment (TME), such MHC-mediated exchange plays a critical role in immune modulation. Acquisition of MHC peptide complexes by tumor or stromal cells can present antigens in a non-canonical context, either sustaining T cell activation or driving T cell exhaustion and tolerance. Conversely, transfer of MHC molecules to regulatory or suppressive immune subsets may dampen effector responses and facilitate immune escape. Thus, trogocytosis-driven MHC transfer constitutes a pivotal mechanism that dynamically reshapes antigen presentation and immune regulation within the TME.

Trogocytosis has been demonstrated to play a dual role in the TME and its outcome is context dependent (Mattei et al., 2022). Tumor cells may exploit trogocytosis to reduce ‘immuno-visibility’, which allows them to adopt a defense mechanism against immune-surveillance. Eventually, by acquisition of inhibitory components, tumor cells can restrict the effective functioning of effector immune-cells (Mattei et al., 2022; Zhao et al., 2022). On the other hand, immune-cells via trogocytosis can restrict the expansion of tumor cells (Mattei et al., 2022). For example, it has been demonstrated that in leukaemia activated NK cells transfer tyrosine-protein kinase receptor (TYRO3), a cell membrane protein of Tumor associated macrophage receptor (TAM) family from tumor cells via trogocytosis. TYRO3^+^NK cells exhibited significantly higher cytotoxicity compared to TYRO3^−^NK cells, thereby showcasing enhanced anti-tumor response (Lu et al., 2021). This bipartite nature makes trogocytosis a ‘double-edged sword’ (Mattei et al., 2022) that is capable of both boosting as well as suppressing immune response to tumors.

Although the mechanism of trogocytosis has been explicitly reported in leukaemia, lymphoma (LeMaoult et al., 2015) and colorectal carcinoma, its broader implications across diverse malignancies are only beginning to be fully appreciated (Delgado-Gonzalez et al., 2022; Singhal et al., 2024; Lehmann et al., 2011; Suzuki et al., 2015). Emerging studies further extend these observations to solid tumors such as renal cell carcinoma (RCC), where tumor cells acquire immune molecules (CD14, CD16, CD56 and CD45) from infiltrating immune-cells (Marcarian et al., 2025), pancreatic ductal adenocarcinoma (PDAC), where cancer-associated fibroblasts transfer cholesterol and plasma membrane lipids to PDAC cells, fostering an immune-suppressive milieu (Ogier et al., 2023) and high-grade serous ovarian carcinoma (HGSC), where trogocytic transfer of CD9 from tumor cells to NK cells hampers their cytotoxicity (Gonzalez et al., 2021). Together, these findings indicate that the broader implications of trogocytosis across diverse malignancies are only beginning to be fully appreciated (Delgado-Gonzalez et al., 2022; Singhal et al., 2024; Lehmann et al., 2011; Suzuki et al., 2015). Malignant tumors are characterized by highly heterogeneous microenvironments, driven by complex interplay of genetic, epigenetic and immunologic factors. Recent studies suggest that trogocytosis contributes not only to immune escape but also to therapy resistance, stemness acquisition, epithelial-mesenchymal transition (EMT) and metastatic spread, all of which are the hallmark features of cancer progression.

This review brings to light the emerging paradigm of trogocytosis as a central yet underexplored mechanism shaping tumor progression and immune landscape across multiple cancer types. By focusing on recent findings, we highlight how trogocytosis orchestrates complex cellular exchanges that not only redefine immune cell function but also drive critical tumor behaviors such as immune evasion, plasticity and resistance. Unlike traditional modes of cell-cell interaction, trogocytosis represents a unique and dynamic conduit of intercellular communication with profound implications for cancer patho-physiology. Recognizing and dissecting this process may unlock novel diagnostic markers and therapeutic strategies applicable across a wide spectrum of malignancies.

## 2 General mechanism of trogocytosis

**FIGURE 1:**
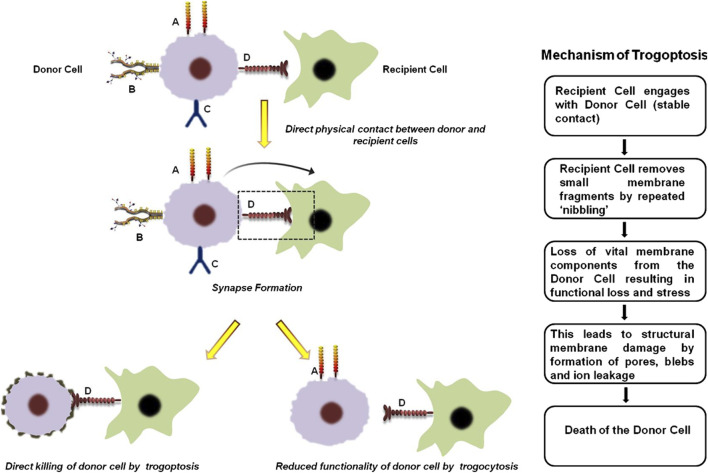
General mechanism of trogocytosis and trogoptosis. The first step of trogocytosis involves the establishment of direct physical contact between the donor and recipient cells. ‘A’, ‘B’, ‘C’ and ‘D’ represents four arbitrary receptors on the donor cell surface that are critical for its normal function. During trogocytosis, a synapse forms between the donor and recipient cells, resulting in the transfer of receptor ‘D’ to the recipient cell. The loss of this receptor significantly impairs the functional capacity of the donor cell. Conversely, acquisition of receptor ‘D’ enables the recipient cell to target the donor cell, leading either to its direct elimination through trogoptosis or to reduced donor cell activity by interfering with receptors ‘B’ and ‘C’. In case of trogoptosis, the recipient cell ‘nibbles’ functional components from the membrane of the donor cell. This results in membrane damage of the donor cell by formation of pores, blebs and ion leakage ultimately leading to cell death (Shin et al., 2021; Toor et al., 2019).

Trogocytosis is a multi-step dynamic process and includes the following events (Figure 1).

1. Direct physical contact between the donor and recipient cells: The first step of trogocytosis involves the initiation of specific receptor-ligand interactions between the two cellular partners. It is almost similar to that observed during immunological synapse formation in immune-cells (Shin et al., 2021).
2. Extraction of membrane fragments: Once the 2 cells are in close proximity, the recipient cell pulls off some portion of the plasma membrane from the donor cell. This results in rapid rearrangement of the cytoskeletal proteins of the plasma membrane resulting in “nibbling” or extraction of various functional, integral and peripheral membrane proteins, lipids and associated cytosolic components. The donor cell thus experiences reduced cellular functionality and sometimes can even undergo trogoptosis (Shin et al., 2021; Toor et al., 2019).
3. Acquisition and expression of the membrane fragments: The recipient cell integrates the acquired cellular fragments from the donor cell into its own plasma membrane. These cells can then either acquire characteristics of the donor cells by functionally expressing the transferred molecules or modulate it is signaling mechanism and interaction with the other cells (Shin et al., 2021; Toor et al., 2019).

## 3 Trogocytosis in cancer

**FIGURE 2:**
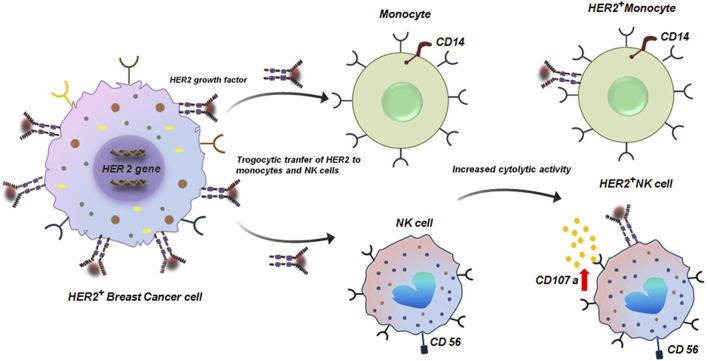
Trogocytic transfer of HER2 to monocytes and NK cells. Transfer of HER protein from HER2^+^ breast tumor cells to CD14^+^ monocytes and CD56^+^ NK cells by trogocytosis. HER2^+^ CD56^+^ NK cells have enhanced cytolytic activity with elevated expression of CD107 (Suzuki et al., 2015).

The phenomenon of trogocytosis in cancer is often considered to be a dynamic and paradoxical process. While the tumor cells exploit this process to escape immune-surveillance and promote metastasis; immune-cells also deploy trogocytosis to exert anti-tumor response. Therefore, gaining a deeper knowledge of these specific mechanisms is essential for creating tailored treatments that either improve anti-tumorigenic trogocytosis in tumor cells or inhibit pro-tumorigenic trogocytosis. For instance, several studies have demonstrated that the complex interplay between breast cancer (BC) cells and the TME involves trogocytic events that eventually direct the course of the disease and therapeutic response (Suzuki et al., 2015). A noteworthy example of trogocytosis in BC involves the HER2 protein. In a study conducted to demonstrate the clinical significance of HER2^+^ tumor-infiltrating immune-cells in HER2^+^ BC patients receiving trastuzumab-based primary systemic therapy (PST), it was revealed that via trogocytosis HER2 can be transferred from BC cells to monocytes and natural killer (NK) cells (Suzuki et al., 2015). Critically, CD107a which is a marker of elevated cytotoxic activity expression is enhanced in trastuzumab-mediated trogocytosed-HER2^+^effector cells (Figure 2). From a clinical perspective, this HER2 expression on tumor-infiltrated immune-cells in HER2^+^ tumors have been correlated with pathological complete response (PCR) to trastuzumab-based PST (Suzuki et al., 2015).

In parallel, Lis et al. showed that stromal Hospicells induce oncologic trogocytosis in BC cells, leading to the expression of N-cadherin and promoting metastatic behavior (Lis et al., 2010). More recently, Sivakoses et al. reported that TNBC cells can acquire CD45 and genomic material from T cells, thereby reducing immunogenicity and enabling immune evasion (Sivakoses et al., 2025).

Importantly, similar patterns have been observed in other malignancies, underscoring the broad oncologic relevance of trogocytosis. In colorectal carcinoma, inhibitory checkpoint molecules such as PD1, Tim3 and CTLA4 are transferred from regulatory T cells to tumor cells, enhancing immune suppression (Betts et al., 2012; Shin et al., 2021). In RCC, tumor cells acquire immune surface proteins such as CD14, CD16, CD56 and CD45 from infiltrating lymphocytes and monocytes, that promotes tumor survival by impairing immune effector function (Marcarian et al., 2025). In PDAC, cancer-associated fibroblasts donate lipids and cholesterol to tumor cells through trogocytosis, establishing an immunosuppressive stroma (Ogier et al., 2023). Similarly, in HGSC, NK-cell cytotoxicity is dampened by the trogocytic acquisition of CD9 from tumor cells (Gonzalez et al., 2021). Collectively, these findings reveal that trogocytosis is a unifying mechanism across multiple cancer types, shaping tumor progression, immune interactions and ultimately therapeutic outcomes.

While these findings underscore the potential of trogocytosis to amplify anti-tumor immunity particularly in enhancing therapeutic efficacy; this phenomenon is not uniformly beneficial. Paradoxically, tumor cells can also co-opt trogocytosis to evade immune-surveillance, remodel the TME and foster disease progression. The following section explores the mechanisms and implications of such pro-tumorigenic trogocytosis, highlighting its role in immune escape and therapeutic resistance.

### 3.1 Pro-tumorigenic trogocytosis

**FIGURE 3:**
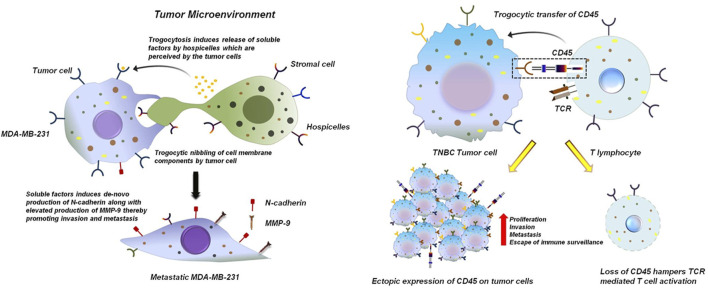
Trogocytosis between tumor cells and stromal cells induces an invasive phenotype to the tumor cells. TNBC breast tumor cells MDA-MB-231 nibbles components from the plasma membrane of hospicelles in the TME. This induces the release of variable soluble factors from the hospicelles. These soluble factors are perceived by MDA-MB-231 which stimulates *de novo* production of N-cadherin by MDA-MB-231. Excess production of N-cadherin bestows the tumor cell with an invasive phenotype and also stimulates the expression of MMP-9, thereby promoting metastasis (Lis et al., 2010; Mehner et al., 2014; Yousef et al., 2014) (left panel). Trogocytosis between TNBC tumor cells and T cells promotes tumor cell proliferation and hampers functionality of T cells. TNBC breast tumor cells siphons CD45 from T lymphocytes. This dampens phosphorylation and activation of TCR preventing its effective functioning. Ectopic expression of CD45 on breast tumor cells helps them to escape the process of immune-surveillance thereby promoting proliferation and invasion of these cells (Sivakoses et al., 2025) (right panel).

In the context of cancer biology, the term pro-tumorigenic refers to cellular and molecular processes that actively favor tumor initiation, progression and survival. Such processes can function through multiple mechanisms, including the enhancement of proliferative and survival pathways in malignant cells, the facilitation of immune evasion, metastasis and angiogenesis and the establishment of a tumor-supportive microenvironment (Banerjee et al., 2014; Goswami et al., 2013; Bhuniya et al., 2020). Within this framework, trogocytosis has emerged as a unique mode of intercellular communication with significant pro-tumorigenic potential. By enabling the direct transfer of membrane-bound receptors, ligands and signaling molecules between tumor cells and immune or stromal cells, trogocytosis can generate functional changes that ultimately support tumor growth. In a study conducted by Raphael et al., in 2010 using BC cell lines it was reported that cancer cells via oncologic trogocytosis can acquire mesenchymal phenotype by interacting with non-tumor cells within the TME (Lis et al., 2010). The study revealed an interesting observation in which MDA-MB-231 cells siphoned patches of cell membrane components from Hospicelles. Hospicelles are stromal cells which were first isolated from ovarian cancer patients. These cells have been reported to be involved in suppressing T-cell functionality as well as conferring chemoresistance by transferring multi-drug resistant proteins (MDR) via trogocytosis to the tumor cells (Lis et al., 2010; Pasquet et al., 2010). Interaction of MDA-MB-231 with Hospicells does not lead to the direct acquisition of mesenchymal molecules. Rather, Hospicells induces production of soluble factors that in-turn lead to the *de novo* expression of mesenchymal proteins specifically N-cadherin (Lis et al., 2010). Expression of N-cadherin confers an invasive phenotype to the BC cells, thereby promoting metastasis. Moreover, N-cadherin increases the sensitivity of tumor cells to fibroblast growth factor 2 (FGF-2), which further enhances the expression of matrix metalloproteinase 9 (MMP-9). MMP-9, being an extracellular matrix (ECM) degrading enzyme plays a predominant role in promoting tumor angiogenesis, invasion and metastasis (Figure 3) (Lis et al., 2010; Mehner et al., 2014; Yousef et al., 2014). Sivakoses et al., 2025 revealed that in triple negative breast cancer (TNBC) patient samples cancer cells siphons CD45 from T-cells via trogocytosis (Sivakoses et al., 2025). CD45 is a receptor-type protein tyrosine and is a crucial player in T-cell receptor (TCR) mediated T cell activation. CD45 activates lymphocyte specific protein tyrosine kinase (LCK) which phosphorylates the TCR complex (Courtney et al., 2019; Bhatta et al., 2020). In case of TNBC, the tumor cells tends to acquire trogosomes which includes CD45 along with T cell genomic DNA. Tumor cells by expressing CD45 and other immune-proteins can escape the process of immune-surveillance by lowering their immunogenicity (Figure 3). This facilitates their proliferation and encourages invasion and metastasis (Sivakoses et al., 2025).

**FIGURE 4:**
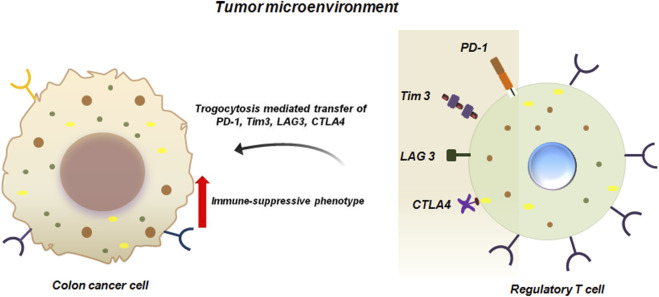
Trogocytosis between colon cancer cells and Tregs promote immune-suppressive functions of cancer cells. Trogocytic interaction between colon cancer cell and Tregs transfers inhibitory molecules like PD1, Tim3, LAG3 and CTLA4 from Tregs to colon cancer cells. This facilitates immune-suppression and enhanced survival of the tumor cells in the TME (Shin et al., 2021).

Apart from BC, trogocytosis has been reported in numerous other malignancies like colon cancer. It has been experimentally proven that hematopoietic cell marker proteins like CD4 and CD45 can be transferred via trogocytosis from tumor infiltrating immune-cells to colon cancer cells (Shin et al., 2021). Importantly, CD4^+^T cells have been demonstrated to be the most abundant lymphocyte population in the TME of murine metastatic colon cancer system. Of these CD4^+^T cells, majority of them are immune-suppressive regulatory T cells (Tregs) which expresses inhibitory molecules like CTLA4, Tim3, PD1 and LAG3. These inhibitory molecules are transferred to colon cancer cells via trogocytosis (Figure 4). Transfer of these immune regulating molecules to the tumor cells enhances the immune-suppressive functions of the cancer cells (Shin et al., 2021).

**FIGURE 5:**
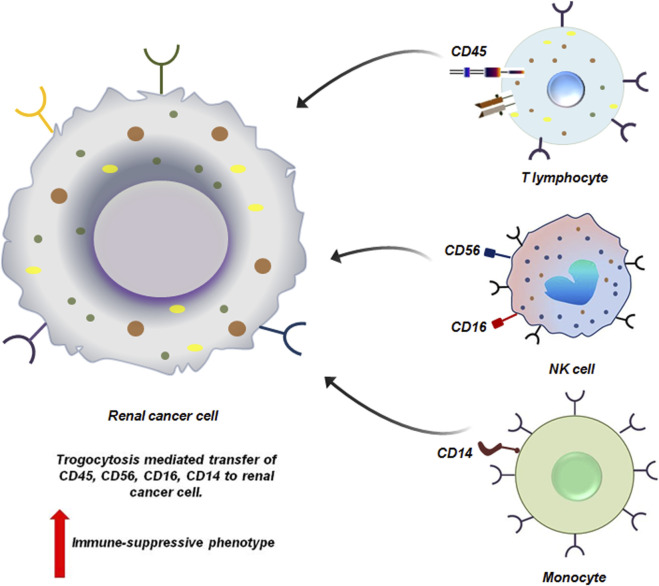
Trogocytosis between renal cancer cell and immune cells induces immune-suppressive phenotype to the cancer cells. Renal cancer cells via trogocytosis acquire different functional molecules from the immune-cells. CD45, CD16 and CD56, CD14 are siphoned from the T lymphocytes, NK cell and monocytes respectively by the tumor cells. Loss of these molecules from the immune-cells affects their effector function severely. This in-turn facilitates the proliferation of the tumor cells by helping them to escape the process of immune-surveillance (Marcarian et al., 2025).

Haley et al. reported the presence of immune markers like CD14, CD16, CD56 and CD45 in RCC patients. These molecules have been transferred from tumor infiltrating immune-cells through trogocytosis (Figure 5). However, it has not yet been elucidated how RCC cells utilizes these acquired immune cell surface proteins to promote their survival (Marcarian et al., 2025). Given the frequency of occurrence of these proteins on RCC cells at all the stages, it may be hypothesized that these molecules do provide survival advantage to RCC cells possibly by evading immune responses (Marcarian et al., 2025).

**FIGURE 6:**
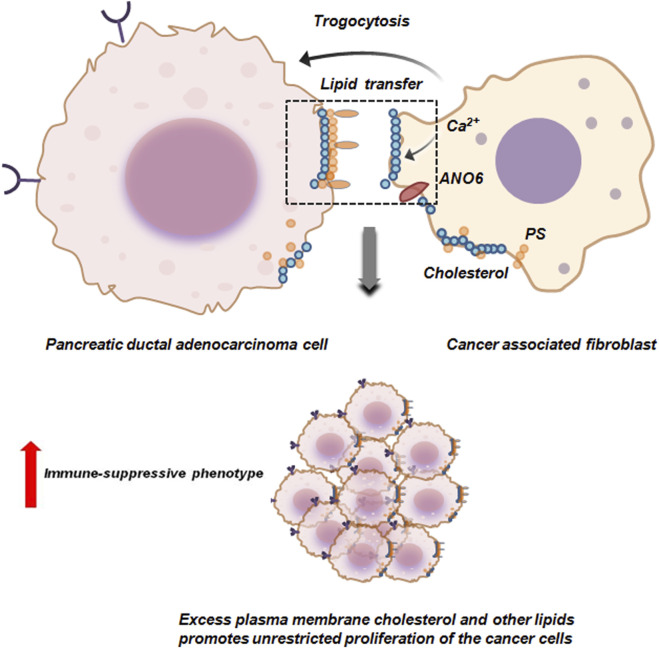
Trogocytosis between pancreatic ductal adenocarcinoma cells and cancer associated fibroblasts facilitates proliferation of the tumor cells. Cancer associated fibroblasts (CAFs) via trogocytosis transfer cholesterol and other plasma membrane lipids to pancreatic ductal adenocarcinoma cells (PDAC). The formation of synapse between PDAC and CAFs induces calcium ion influx in CAFs which in-turn activates anoctamin 6 (ANO6) scramblase. ANO6 facilitates externalization of phosphotidyl serine (PS) on the plasma membrane of CAFs. This initiates the trogocytic transfer of membrane lipids like cholesterol to PDAC cells thereby promoting immune-suppression and survival by unrestricted proliferation of PDAC cells (Ogier et al., 2023).

In PDAC trogocytosis supports the survival of PDAC cells in fibroblastic, avascular stroma by providing essential metabolites and lipids. Cancer associated fibroblasts (CAFs) supply lipids from blood borne particles to PDAC cells by plasma membrane trogocytosis. During trogocytosis, PDAC cells and CAFs form synapse which induces calcium ion influx. This influx stimulates phospholipid scramblase anoctamin 6 (ANO6) resulting in exposure of phosphotidyl serine (PS) on CAFs plasma membrane. This initiates the trogocytic transfer of membrane lipids like cholesterol to PDAC cells (Figure 6). Excessive transfer of cholesterol on the other hand is associated with an immune-suppressive milieu thereby supporting the unrestricted growth of PDAC cells (Ogier et al., 2023).

**FIGURE 7:**
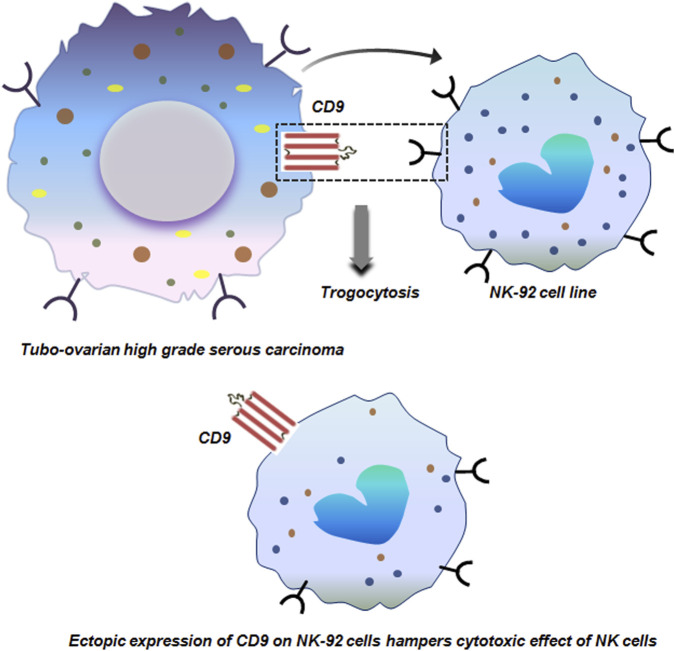
Trogocytosis between tubo-ovarian high grade serous carcinoma and NK-29 cells. Trogocytic transfer of CD9 from tubo-ovarian high grade serous carcinoma to NK-29 cells dampens the cytotoxic effect of NK cells with reduced production of anti-tumor cytokines (Gonzalez et al., 2021).

Tubo-ovarian HGSC is an extremely lethal gynecological malignancy due to its advanced stage diagnosis, making it quite challenging to target. Inspite of the promising role of immunotherapy in cancer treatment, HGSC tumors have not shown any clinical benefit in immunotherapy. A deeper analysis of the HGSC TME revealed that, apart from exhausted T cells, decidual like NK (dNK) cells (CD56^+^CD9^+^CXCR3^+^KIR^+^CD3^−^CD16^−^) are prevalent in the HGSC TME. These dNK cells are distinct from the NK cells and correlates with the abundance of HGSC tumor. *In-vitro* co-culture of NK-92 cell line with HGSC cells resulted in trogocytic transfer of CD9 from the tumor cells to NK cells. This acquisition of CD9 hampered the cytotoxic activity of NK cells with reduced production of anti-tumor cytokines (Figure 7). Targeting CD9 with blocking antibody or with clustered regularly interspaced short palindromic repeats (CRISPR) restored the cyto-toxicity of NK cells. This demonstrates the causal role of trogocytosed CD9 in dampening NK-cell anti-tumor responses (Gonzalez et al., 2021).

**FIGURE 8:**
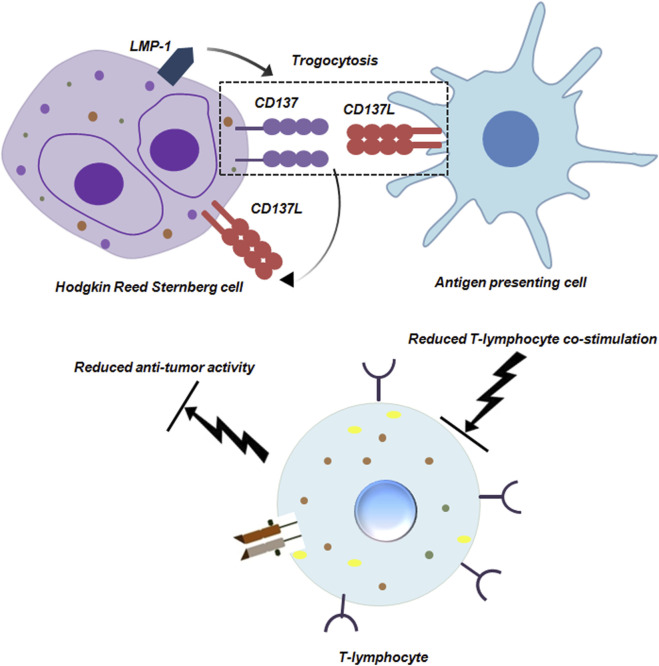
Trogocytosis between Hodgkin Reed Sternberg Cell and Antigen presenting cell reduces anti-tumor activity of T-lymphocytes. Hodgkin Reed Sternberg (HRS) cells ectopically express CD137 induced by LMP-1. CD137 can bind to CD137L on the HRS cell as well as on antigen presenting cells (APCs). The interaction between CD137 and CD137L on HRS and APCs respectively is mediated by trogocytosis. Engagement of APCs in trogocytosis hampers their co-stimulatory ability, dampening anti-tumor functionality of T-lymphocytes (Zeng and Schwarz, 2020).

Trogocytosis is not only restricted to carcinomas. It is also prevalent in other cancers like Hodgkin lymphoma (HL). An extremely characteristic feature of HL is the presence of Hodgkin and Reed Sternberg (HRS) cells. HRS cells are large, mono or multinucleated and constitute 1% of the entire tumor mass. HRS cells can efficiently escape the process of immunesurveillance by trogocytosis (Zeng and Schwarz, 2020; Yadav et al., 2022). An important molecule in this regards is CD137. Interaction between CD137 and CD137L polarizes T cells to T helper 1 cell (Th1) type by transducing co-stimulatory signal to the T cells. This polarization of T cells is extremely pivotal for anti-tumor immunity. To hamper this anti-tumor activity, HL cells ectopically express CD137 transduced by LMP-1 (latent membrane 1 protein). This ectopic expression of CD137 performs two distinctive roles. It can bind to CD137L on the HRS itself and cause internalization of this protein. It can also bind to CD137L on surrounding antigen-presenting cells (APCs) by trogocytosis and downregulate CD137 thereby leading to dampening of co-stimulation of T cells (Figure 8; Zeng and Schwarz, 2020).

**FIGURE 9:**
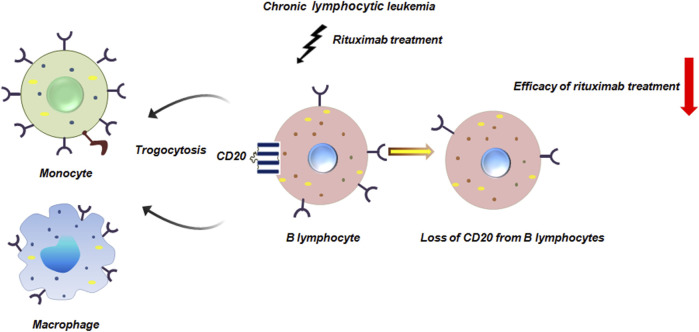
Trogocytosis in chronic lymphocytic leukemia reduces efficacy of rituximab. Rituximab is a commonly used monoclonal antibody in chronic lymphocytic leukemia treatment. Rituximab infusion leads to significant loss of CD20 from B lymphocytes by monocytes or macrophages mediated by trogocytosis thereby compromising the effectiveness of rituximab treatment (Beum et al., 2006).

In chronic lymphocytic leukemia infusion of rituximab induces significant loss of CD20 from circulatory B lymphocytes by monocytes or macrophages via trogocytosis (Beum et al., 2006). The Fcγ receptors on these immune-cells bind to the rituximab-CD20 complex on B cells. The macrophages then internalize the complex, effectively stripping the CD20 and bound rituximab from the B cell surface without killing it. From early pre-B to mature B cell stages, CD20, a B cell differentiation antigen, is widely expressed. However, it is eliminated as B cells differentiate into plasma cells (Pavlasova and Mraz, 2020; Dabkowska et al., 2024). This trogocytosis-mediated removal of CD20 not only reduces the efficacy of rituximab therapy but also contributes to immune escape by diminishing the targetable antigen density on leukemic cells (Figure 9).

**FIGURE 10:**
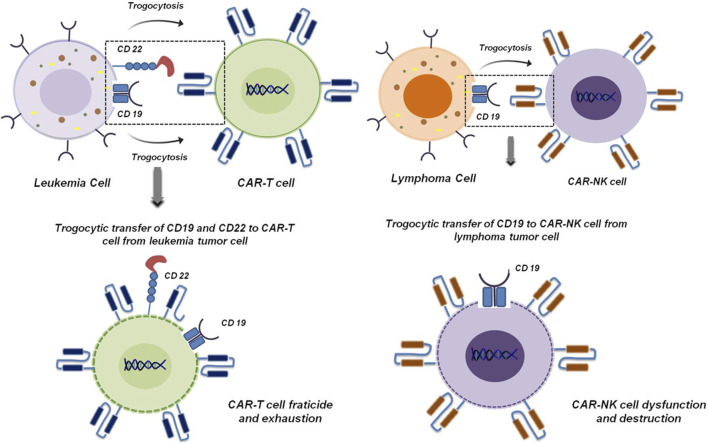
Trogocytosis mediated fracticide and dysfunctioning of CAR-T and CAR-NK cells. Transfer of CD19 and CD22 from leukemia tumor cells to CAR-T cells induces CAR-T cell fratricide and exhaustion (Miyake and Karasuyama, 2021; Vander Mause et al., 2022) (left panel). In lymphoma, ectopic expression of CD19 mediated by trogocytosis induces CAR-NK cell dysfunction and destruction (Martinez-Martin and Alarcon, 2023) (right panel).

Besides the known traditional role of trogocytosis in cancer, its importance is increasingly recognized in chimeric CAR immune cell mediated therapy (Chen et al., 2024). Amidst significant advances in cancer treatment modalities, CAR-based immunotherapy has garnered substantial prominence as powerful complement to conventional treatment strategies due to recent breakthroughs in cancer research. Although it has shown remarkable potential, accumulating clinical evidence and patient follow up data underscore tumor relapse as a major obstacle, with trogocytosis emerging as a key contributing factor (Chen et al., 2024). Trogocytosis-mediated tumor immune escape has been demonstrated in CAR-T and CAR-NK therapy studies. It has been reported in leukemia that tumor cells can transfer target specific antigens to the surface of CAR-T and CAR-NK cells via trogocytosis. This dampens the expression of antigen on the surface of tumor cells (Chen et al., 2024; Miyake and Karasuyama, 2021). A prominent example of this is the transfer of CD19 from leukemia cells to CAR-T cells mediated by trogocytosis (Miyake and Karasuyama, 2021). This ultimately leads to CAR-T cell fratricide and exhaustion. A similar phenomenon has also been reported in acute lymphoblastic leukemia in which CD22 is trogocytosed to CAR-T cells from tumor cells leading to their destruction (Figure 10) (Vander Mause et al., 2022). In lymphoma, ectopic expression of CD19 on CAR-NK cells promotes its dysfunctioning and destruction (Figure 10) (Martinez-Martin and Alarcon, 2023). On the other hand, it has been reported that trogocytosis drives CAR loss and impairs T cell function. Aberrant CAR expression on tumor cells masks antigens, fueling immune escape and resistance (Chen et al., 2024).

### 3.2 Anti-tumorigenic trogocytosis

**FIGURE 11:**
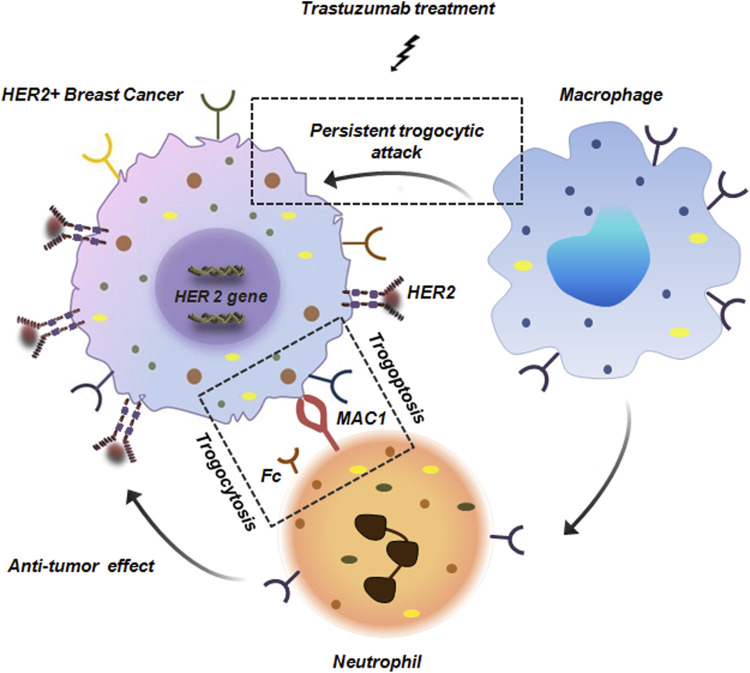
Anti-tumor trogocytosis in HER2^+^ breast cancer. Persistant trogocytic attack from macrophages results in effective reduction of HER^+^ cells upon treatment with trastuzumab. Along with macrophages, neutrophils with Fc receptors are also capable of interacting with HER^+^ cells. These neutrophils express MAC1 integrin that facilitates its binding with the tumor cells thereby initiating trogocytosis and anti-tumor response (Matlung et al., 2018; Chen et al., 2025a).

Trogocytosis is a multi-faceted cellular process. While tumor cells exploit pro-tumorigenic trogocytosis to escape the process of immune-surveillance, immune-cells such as NK cells, T cells and macrophages actively engage in anti-tumorigenic trogocytosis. These involve mechanisms like acquisition of tumor antigens which enhances immune recognition and priming and can even lead to direct death of tumor cells via trogoptosis. Study conducted by Velmurugan et al. demonstrated that macrophages via persistant trogocytic attack can effectively reduce 50% of antibody opsonized HER over-expressing BC cells over a span of 3 days (Martinez-Martin and Alarcon, 2023; Velmurugan et al., 2016). Co-culture of macrophage cell lines like J774A.1 or RAW264.7 with MDA-MB-453 or SKBR3 cells in presence of trastuzumab resulted in 90% and 50% reduction in cells respectively (Martinez-Martin and Alarcon, 2023; Velmurugan et al., 2016). Along with macrophages, in BC, netrophils have also been reported to show anti-tumor effects. Although, neutrophils lack the intrinsic ability to phagocytose tumor cells but neutrophils armed with Fc receptors can eliminate tumor cells by antibody-dependent cellular cytotoxicity (ADCC) (Matlung et al., 2018). In BC, neutrophils extract cell membrane components from tumor cells mediated by trogocytosis which leads to tumor suppression. Moreover, this interaction between neutrophils and BC is facilitated by Macrophage-1 antigen (MAC-1) integrin which facilitates trogoptosis of BC cells (Figure 11; Matlung et al., 2018; Chen et al., 2025a).

In melanoma and myeloma it has been observed that CD8^+^T cells derived from peripheral blood mononuclear cells (PBMCs) take up membrane components from autologous melanoma cells or melanoma cell lines upon direct contact (Dionne et al., 2001; Zhou et al., 2005). This membrane acquisition is dependent on T cell receptor (TCR) engagement and is specific to certain epitopes, occurring predominantly in highly cytotoxic T cell clones (Machlenkin et al., 2008). When these membrane capturing CD8^+^T cells were transferred into mouse models bearing human melanoma tumors, they effectively suppressed tumor growth. It has further been demonstrated that both CD4^+^ and CD8^+^ T cells found in the PBMCs and tumor-infiltrating lymphocytes (TILs) of melanoma patients are capable of capturing melanoma antigens, leading to enhanced tumor-specific reactivity and cytotoxicity (Eisenberg et al., 2013).

**FIGURE 12:**
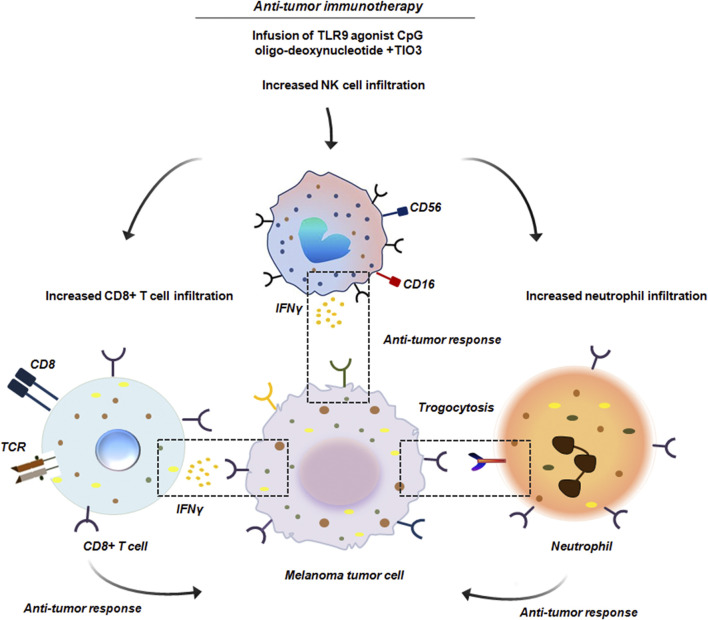
Anti-tumor immunotherapy in B16 melanoma. Administration of anti-tumor immunotherapy by peritoneal injection of the TLR9 agonist CpG oligodeoxynucleotide combined with TGF-β2 inhibitor TIO3 promoted infiltration of neutrophils within the tumor nodules. These neutrophils by trogocytosis initiated anti-tumor response. Along with neutrophils, a significant infiltration of CD8^+^ T cells and NK cells was also noted. These cells produced IFNγ and induced an effective anti-tumor activity (Zhu et al., 2024).

In B16 melanoma model it has been observed that neutrophils recruited to initial tumor nodules, formed following subcutaneous injection of B16 melanoma cells, were capable of attacking tumor cells through trogocytosis. Further it has been shown that administration of anti-tumor immunotherapy specifically, peritoneal injection of toll like receptor 9 (TLR9) agonist CpG oligodeoxynucleotide combined with the TGF-β2 inhibitor TIO3 enhanced the presence of trogocytic neutrophils within the tumor nodules. This treatment also increased the infiltration of CD8^+^ T cells and NK cells, along with their production of interferon (IFN)-γ (Figure 12). Conversely, local administration of Cxcl2 small interfering RNA (siRNA) led to a marked decrease in both total and trogocytic neutrophils, as well as a reduction in CD8^+^ T and NK cells. This in-turn resulted in the formation of larger tumor nodules thereby confirming the pivotal role of neutrophils in TME (Zhu et al., 2024).

**FIGURE 13:**
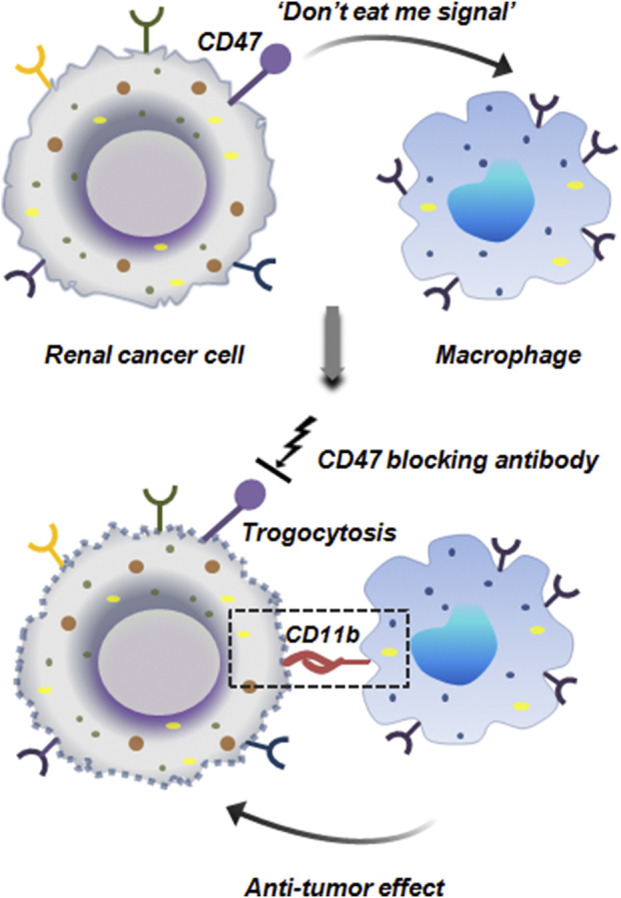
Anti-tumor trogocytosis in renal cancer. Renal cancer cells ectopically express CD47 which inhibits the phagocytic activity of macrophages by sending a ‘do not eat me signal’. Blocking of CD47 restores the anti-tumor activity of macrophages. Macrophages by expressing CD11b integrin binds to the tumor cells and initiates trogocytosis mediated cellular elimination (Park et al., 2022).

In RCC it has been experimentally proven that RCC cells tend to hijack the CD47 signaling mechanism to prevent macrophage mediated elimination. CD47 is an important checkpoint molecule like PD-1/PD-L1 that conveys ‘do not eat me signal’ to the macrophages thereby preventing phagocytosis. Mechanistically, CD47 binds to SIRPα on macrophages which in the downstream targets the phagocytic process. RCC cells by overexpressing CD47 prevent macrophages from attacking them. Interestingly, when CD47 expression was hindered by CD47 blocking antibody, it enhanced anti-tumor effect of macrophages against RCC cell lines. This CD47 blockage induced trogocytosis which enhanced the cell to cell contact between macrophages and RCC cell lines via CD11b integrin signaling in macrophages. Confocal microscopy and live imaging revealed that CD47 blockade increased macrophage-tumor cell contact in terms of both frequency and duration, facilitating more trogocytic events. Further, macrophages with higher CD11b expression exhibited stronger CD47-blockade induced trogocytosis (Figure 13). Thus CD11b apart from its established role as an important integrin, also facilitates trogocytosis (Park et al., 2022).

**FIGURE 14:**
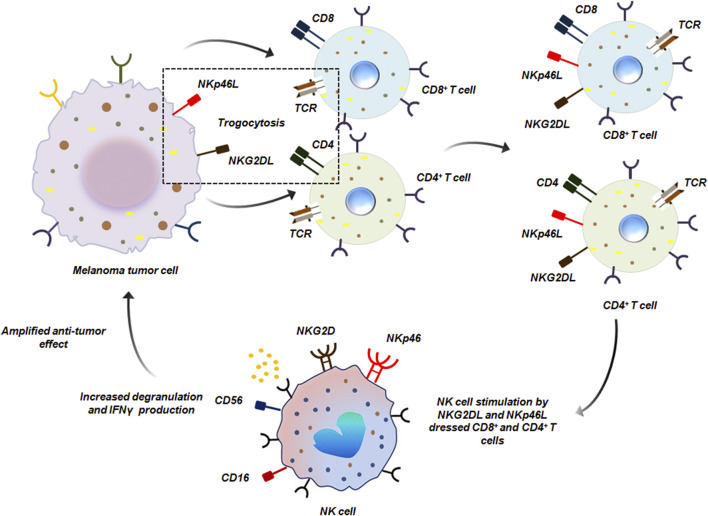
NK cell and T cell mediated anti-tumor trogocytosis. CD8^+^ and CD4^+^ T cells acquire NKG2DL and NKp46L from melanoma tumor cells by trogocytosis. Such ‘dressed’ T cells are recognized by NK cells which bind to NKG2DL and NKp46L by NKG2D and NKp46 receptors respectively on its surface thereby initiating anti-tumor response (Domaica et al., 2009).

Trogocytosis has also been reported to encourage the crosstalk between innate and adaptive immune-cells to ensure anti-tumor immunity. For instance, both CD4^+^ as well as CD8^+^ T cells upon encounter with melanoma tumor cells, capture NKG2D (natural killer group 2, member D) and NKp46 (natural killer cell receptor 46) ligands by trogocytosis from the tumor cells. T cell ‘dressing’ with tumor derived ligands enables them to effectively stimulate NK cells. When NK cells encounter these ‘dressed’ T cells, there was a marked increase in degranulation and IFN-γ secretion, mediated via NKG2D and NKp46 engagement. This effectively amplified the immediate immune responses against melanoma by NK cells induced by T cells (Figure 14) (Domaica et al., 2009).

### 3.3 Trogocytosis and stem cells

While trogocytosis is commonly associated with immune and tumor cells, it is also performed by mesenchymal stem cells (MSCs). MSCs migrate towards the tumor, following a gradient of growth factors, chemokines and inflammatory cytokines released within the TME. This tropism is mediated by factors such as stromal cell derived factor-1 (SDF-1/CXCL12), vascular endothelial growth factor (VEGF), platelet-derived growth factor (PDGF) and transforming growth factor-β (TGF-β), which collectively recruit MSCs to areas of tissue remodeling and malignancy (Melzer et al., 2018). Within the TME, MSCs engage in direct interactions with tumor cells via paracrine signaling and exchange of soluble mediators, which affects the course of cancer, immune evasion and the likelihood of metastasis. Along with these well-established interactions mediated by growth factors and cytokines, new research shows that MSCs can also participate in physical contact-dependent processes like trogocytosis, which allows the transfer of genetic material and functional surface molecules to tumor cells (Marcarian et al., 2025). All of these results point towards a complex interplay between MSC and tumors, where immune regulation and tumor formation are influenced by both direct cell-cell contact as well as soluble factor signaling.

**FIGURE 15:**
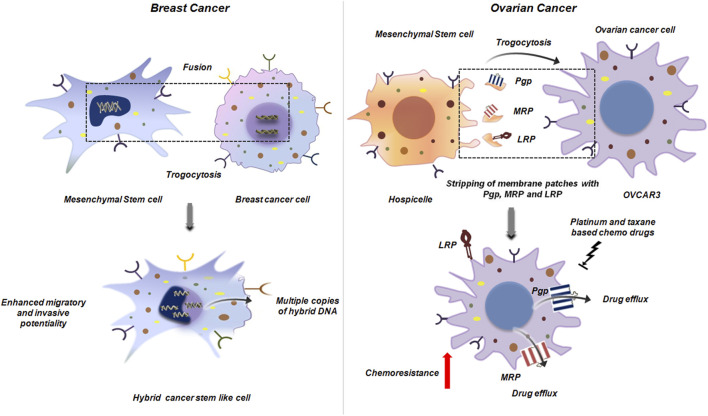
Trogocytosis between mesenchymal stem cell and cancer cell. Mesenchymal stem cell interacts with breast cancer cell to generate a hybrid cancer stem like cell by trogocytic fusion. This hybrid cell has multiple copies of altered DNA, mixed gene expression and heightened metastatic potential (Melzer et al., 2018) (left panel). In ovarian cancer, trogocytosis between hospicells and OVCAR3, results in stripping of the plasma membrane from hospicells with the major ATP dependent drug efflux pumps like Pgp, MRP and LRP. Acquisition of these molecules by OVCAR3 results in outflow of the therapeutic drugs that augments chemoresistance. This further reduces the efficacy of platinum and taxane based chemotherapeutic drugs thereby posing a major challenge in ovarian cancer treatment (Rafii et al., 2008) (right panel).

When MSCs come into direct contact with tumor cells, trogocytosis can lead to cellular fusion, giving rise to hybrid cancer cells. These hybrids possess altered structural and functional traits of predominantly either parental cell (Melzer et al., 2018). In BC, such cellular fusion results in hybrids with heightened DNA ploidy, mixed gene expression profile and enhanced migratory and metastatic potentialities compared to the parental population (Melzer et al., 2018; Noubissi et al., 2015; Rappa et al., 2012; Noubissi and Ogle, 2016). It may be possible that these hybrid cells have enhanced expression of major metastasis promoting factors like Vimentin, Twist, Snail and Slug. Also such hybrids can exhibit cancer stem cell like invasive properties which again is a serious challenge to cancer treatment (Figure 15) (Melzer et al., 2018). In another study by Yang et al., in 2014, using different human ovarian cancer cell lines it was revealed that direct physical contact between MSCs and tumor cells promoted proliferation of the tumor cell through exchange of membrane components by oncologic trogocytosis (Yang et al., 2015; Melzer et al., 2016).

Hospicelles as mentioned earlier are stromal cells which were isolated from the ascitis of ovarian cancer patients (Rafii et al., 2008). Extensive studies on these cells have lead to the conclusion that these cells represent differentiated stromal sub-set of the mesenchymal stem cells that express unique set of markers on their surface. These cells are positive for CD9, CD10, CD29, CD146, CD166 and MDR. Among the MDR proteins Hospicelles extensively express P-glycoprotein (Pgp) family proteins like Pgp, multi-drug resistant protein (MRP), breast cancer resistant protein (BCRP) and lung resistant protein (LRP). These cells exhibit strong, preferential adhesion to epithelial ovarian cancer cells (e.g., OVCAR3) compared to other fibroblasts or endothelial cells. Through this interaction, ovarian cancer cells gain chemoresistance to platinum and taxane-based therapies, largely via transfer of MDR proteins. The cancer cells through distinct mechanism of trogocytosis strip patches of membrane from Hospicells, thereby acquiring functional membrane proteins like Pgp and LRP and concomitantly augmenting the drug resistant phenotype (Figure 15) (Rafii et al., 2008).

## 4 Therapeutic implications of trogocytosis

Trogocytosis may be exploited for therapeutic purposes, particularly as a mechanism for targeted drug delivery. Since immune-cells can acquire membrane-bound molecules through direct cell contact, this process may be co-opted to deliver cytotoxic agents, immune-modulating ligands or signaling payloads to malignant cells. One proposed strategy involves engineering immune-cells to carry surface bound drugs or nanoparticles, which are then transferred to tumor cells via trogocytosis in a spatially restricted and antigen-specific manner. This cell-to-cell delivery approach may improve the therapeutic index by concentrating activity at the tumor site while limiting systemic exposure and toxicity. Moreover, given the directional nature of trogocytosis, such methods could allow for the precise targeting of metastatic niches or tumor stroma interfaces that are often difficult to reach with conventional therapies (Chen et al., 2025b).

While the therapeutic potential of trogocytosis is promising, a clearer roadmap for its clinical translation is still emerging. Table 1 highlights representative clinical trials, mechanistic insights and potential solutions that could accelerate the incorporation of trogocytosis into practice.

**TABLE 1:** Ongoing or recent clinical studies and protocols involving trogocytosis [ClinicalTrials.gov].

Sl. No	Short title/Sponsor	Status	How trogocytosis is involved	Clinicaltrials. Gov ID	Potential Solution
1	Is Trogocytosis a Predictive Marker of CAR-T Cell Response in Diffuse Large B-cell Lymphoma?	Enrolling	Study explicitly aims to identify trogocytosis signature on immune cells as a predictor of clinical response after CAR-T infusion	NCT06352242	Embed standardized trogocytosis assays (flow/CyTOF) in CAR-T monitoring; use results to guide patient selection or early intervention
2	A Phase 2, Open-Label Study to Evaluate the Safety, Efficacy and Pharmacokinetics of Amivantamab Monotherapy in Participants with Previously Treated Advanced Hepatocellular Carcinoma	Active/Phase 2	The investigator brochure and protocol explicitly discuss ADCT (antibody-dependent cellular trogocytosis) as one of the Fc-dependent mechanisms of action and a read out of effector engagement	NCT05653427	Fc-engineering to optimize ADCT; combination with checkpoint inhibitors to amplify effector function
3	Amivantamab With Tyrosine Kinase Inhibitors (TKI) for Advanced NSCLC With ALK, ROS1, or Ret alterations	Active/Recruiting	Protocol text and trial description note trogocytosis/ADCT among Fc effector mechanisms being investigated in biomarker and mechanistic substudies	NCT05845671	Pair with immune-stimulatory agents to enhance trogocytic cytotoxicity while limiting off-target effects

Trogocytosis also offers an emerging platform for biomarker discovery and immune monitoring. During immune-tumor interactions, immune-cells may acquire tumor-specific molecules, including tumor antigens, checkpoint molecules (e.g., PD-1, PD-L1) (Hasim et al., 2022; Gary et al., 2012; Kawashima et al., 2021) or other surface markers, providing a molecular fingerprint of recent antigen engagement. These trogocytosed markers can be detected using advanced single-cell platforms such as high-parameter flow cytometry, mass cytometry (CyTOF) and spatial transcriptomics enabling precise mapping of cellular interactions within the tumor microenvironment. For example, the detection of PD-1 on NK cells has been proposed as an indirect marker of contact with PD-1 expressing tumor cells and may serve as a biomarker for response to checkpoint blockade therapy (Hasim et al., 2022). Furthermore, trogocytosed molecules found in circulating immune-cells or extracellular vesicles could represent a novel class of liquid biopsy biomarkers, offering non-invasive tools for real-time monitoring of therapeutic efficacy, immune engagement and resistance mechanisms. These translational advances underscore the potential of trogocytosis in diagnostics and precision oncology.

While the mechanistic aspects of trogocytosis are central to understanding therapeutic resistance, its translational significance must also be viewed through patient-centered outcomes. Relapse following CAR-T therapy or monoclonal antibody treatment due to trogocytosis mediated antigen loss not only reduces survival but also profoundly impacts quality of life. Repeated lines of therapy expose patients to cumulative toxicities, fatigue, financial strain and loss of work productivity, all of which enhances the disease burden. Moreover, the psychological stress of recurrence particularly after initial remission can exacerbate distress, anxiety and diminish patients trust in emerging therapies. Recognizing these patient experiences underscores the urgency of developing interventions that not only prolong survival but also safeguard long-term quality of life.

From an ethical perspective, many of the strategies designed to circumvent trogocytosis like armored CAR constructs, trogocytosis inhibitors or biomarker guided adaptive therapies are resource intensive. This raises concerns about equitable access, especially in low and middle income settings where advanced immunotherapies remain scarce. Integrating affordability and accessibility into the design and deployment of trogocytosis targeted strategies is therefore crucial to ensure that breakthroughs do not inadvertently widen disparities in cancer care.

Therefore, future research must not only refine the mechanistic and therapeutic manipulation of trogocytosis but also incorporate patient-centered outcomes. Long-term follow-up studies assessing relapse rates, durability of remission and post-treatment toxicities will be essential to evaluate whether targeting trogocytosis translates into tangible improvements in survival and survivorship.

## 5 Emerging ethical challenges

As the clinical translation of trogocytosis targeted approaches accelerates, several ethical challenges warrant careful consideration. First, the very mechanisms that make trogocytosis therapeutically exploitable such as antigen acquisition, immune modulation and membrane transfer carry the inherent risk of unintended immune suppression or off-target effects. For example, genetically engineered immune cells designed to enhance trogocytosis might inadvertently strip antigens from healthy cells, compromising normal immune-surveillance or inducing autoimmunity. Similarly, trogocytosis based delivery of therapeutic molecules could result in unanticipated cross-talk between non-malignant cell types within the TME leading to unpredictable clinical outcomes. Second, the advent of engineered constructs such as armored CAR-T or CAR-NK cells highlights the ethical responsibility to ensure robust preclinical safety testing. Genetically modified effector cells carry risk of unrestrained activity, clonal expansion or long-term immune dysregulation. Rigorous monitoring frameworks and adaptive trial designs will therefore be essential to minimize side effects while optimizing therapeutic benefit.

Finally, patient autonomy and informed consent represent pivotal ethical priorities. Given the novelty and complexity of trogocytosis targeted interventions, patients must be fully apprised of both potential benefits and risks, including uncertainties surrounding long-term outcomes. Clear communication of risks such as therapy resistance, relapse or unintended immune suppression is critical for building trust and ensuring ethical conduct of clinical trials. Hence, addressing these emerging ethical challenges proactively through transparent trial designs, equitable access policies and patient centered communications will be central to responsibly advancing trogocytosis from mechanistic insight to a clinically viable therapeutic strategy.

## 6 Discussion

Trogocytosis has emerged as a pivotal mechanism in malignancy, with dualistic roles that influence tumor progression, immune regulation and therapeutic outcomes. Though some aspects are well-characterized, numerous mechanistic and translational questions remain unanswered. Recent literature highlights trogocytosis’s contrasting roles. On one hand, tumor-infiltrating lymphocytes (TILs) can transfer surface proteins to cancer cells such as CTLA-4, PD-1, LAG-3, TIM-3, VISTA, CD38, CD80, CD86, MHC II and PD-L1 leading to an immunosuppressive TME and dampened anti-tumor immune activity (Kim et al., 2025; Suzuki et al., 2015; Shin et al., 2021; Pagliano et al., 2022; Li et al., 2021).

Trogocytosis can also confer antigenic modulation leading to therapeutic resistance. Many antibody therapies (e.g., rituximab targeting CD20) are subjected to antigen ‘shaving’ where trogocytosis by FcR^+^ cells removes therapeutic targets, diminishing efficacy (Beum et al., 2006). For CAR-T cell therapies, trogocytosis-mediated loss of antigen from target cells (and fratricide of trogocytic T cells) limits persistent antitumor activity (Domaica et al., 2009).

Conversely, trogocytosis can bolster immune responses. CD8^+^ T cells exhibit trogocytic ‘nibbling’ of antigen-presenting cells (APCs), leading to antigen re-presentation, enhanced cytokine secretion and proliferation (Pagliano et al., 2022). Although, this may also induce fratricide or exhaustion. NK cells similarly acquire tumor antigens or co-stimulatory receptors like NKp46 and NKG2D via trogocytosis, potentially enhancing cytotoxicity and tumor-homing capacity (Domaica et al., 2009; Ramezani et al., 2023). Macrophages, via antibody-dependent trogocytosis, can not only strip cancer cell targets but also induce cell death by trogoptosis showing potential as a complementary therapeutic mechanism (Velmurugan et al., 2016).

Insight into mechanisms controlling trogocytosis opens avenues to regulate this process therapeutically. The Cholesterol-25-Hydroxylase (CH25H) pathway, which modulates cholesterol in the membrane, suppresses trogocytosis (Lu et al., 2022). Mechanistically, CH25H converts cholesterol to 25-hydroxy-cholesterol (25-HC) which effectively inhibits trogocytosis and prevents fratricide of cytotoxic T lymphocytes. However, downregulation of CH25H by tumor conditioned media correlates with increased trogocytosis and diminished T cell killing capacity. Over-expressing CH25H in CAR-T constructs (‘armored CARs’) restores killing efficiency, reduces exhaustion and extends survival in mouse models (Lu et al., 2022). Novel strategies to degrade acquired inhibitory molecules hold promise, such as Siglec-7/9 degraders by inhibiting trogocytosis restore T cell function and synergize with checkpoint inhibitors in preclinical models (Wang, 2025). Targeting trogocytic suppressors like Tim-3, which mediates T cell acquisition of myeloid proteins and subsequent fratricide, may enhance dendritic cell (DC)-mediated antitumor activity (Pagliano et al., 2022).

In addition to reshaping immune interactions, trogocytosis exerts profound influence on stemness, EMT and metastatic dissemination. Notably, trogocytosis with stromal or MSCs can reprogram cancer cell phenotype towards greater plasticity and invasiveness. EMT is tightly linked with stemness; a phenotype characterized by self-renewal, therapy resistance and metastatic potentialities (Melzer et al., 2018; Noubissi et al., 2015; Rappa et al., 2012; Noubissi and Ogle, 2016). Trogocytosis mediated induction of EMT markers, such as N-cadherin and Vimentin, likely fosters a mesenchymal state that confers stem-like behavior and therapy resilience. This suggests that trogocytosis may serve as an additional route to enhance tumor cell plasticity and metastatic potential (Lis et al., 2010).

Despite the rapid progress, trogocytosis in cancer remains partly enigmatic, with several critical under-explored areas. Although trogocytosis is recognized as a mechanism driving EMT and stemness, detailed molecular pathways such as the identity of soluble EMT inducers and the downstream signaling networks remain to be elucidated. Exploring how trogocytosis interfaces with EMT promoting transcription factors (e.g., Snail, Twist, ZEB) and with niche elements like platelets or stromal cells could reveal actionable entry points.

Along with EMT, identifying molecular regulators of trogocytosis in cancer cells, immune-cells and stromal components is essential. Molecules such as ICAM-1, CH25H and ATF3 are implicated, but comprehensive mechanistic pathways remain unresolved. The question of what determines the repertoire of molecules transferred (co-stimulatory vs. inhibitory) needs more investigation. Further, it is also not known whether trogocytosis preferentially occur in specific cancer sub-clones or at any particular disease stages. Evidence in colorectal cancer suggests greater occurrence in mutated, late-stage cells along with aggressive onco-mutations (APC, KRAS, TP53, SMAD4) (Shin et al., 2021). However, it cannot be concluded if this is a universal occurrence in all malignancies.

Recent advances from deciphering its role in immune regulation and antigen presentation to engineering CAR constructs and developing selective degraders highlights that precise modulation of trogocytosis holds strong promise in cancer therapy. Nonetheless, the field requires comprehensive mechanistic frameworks, patient-based evaluations and integrated strategies combining biomarker monitoring with targeted interventions. This multi-pronged path will determine whether trogocytosis evolves from a nuance of cell biology into a cornerstone of next-generation oncology. By exploring the molecular determinants, clinical relevance and therapeutic manipulability of trogocytosis, future research can convert this under-appreciated process into an actionable axis within precision oncology moving from ‘nibble’ and ‘evade’ to ‘target’ and ‘triumph’.

## 7 Conclusion

In-spite of significant advances, several critical questions remain that will determine the successful translation of trogocytosis into clinical oncology. A major gap is in defining the molecular signals that dictate whether trogocytosis will favor tumor growth or immune suppression *versus* anti-tumor responses. Current evidence points towards the importance of tumor-initiating receptor-ligand interactions, e.g., FcγR-antibody complexes, TCR-MHC engagement (Miyake and Karasuyama, 2021), CD47-SIRPα signalling (Park et al., 2022), cell intrinsic regulators such as CH25H mediated cholesterol metabolism and ATF3 transcriptional control within the TME (Lu et al., 2022). However, the relative contribution of these factors and their integration into a unified regulatory network remains unresolved. Deciphering these hierarchies will likely require approaches such as CRISPR, single-cell spatial multi-omics and live-cell imaging of immune-tumor synapses in both animal model systems and patient-derived samples.

Next, an area of urgent investigation is the development of strategies to selectively modulate trogocytosis without compromising systemic immune function. While inhibition of pro-tumorigenic trogocytosis or enhancement of anti-tumor responses holds therapeutic promise, indiscriminate suppression of trogocytosis could weaken essential immune-surveillance against pathogens or interfere with normal intercellular communication. Potential solutions include engineering antigen receptors that preferentially act in high antigen density tumors, designing localized or transient modulators of trogocytosis pathways and incorporating inducible safety switches into engineered cell therapies to minimize long term risks. Preclinical validation of such strategies must rigorously assess systemic immune competence, using infection models, vaccine response assays and longitudinal profiling of immune repertoires.

Finally, robust clinical application of trogocytosis research will depend on the ability to monitor this process in real time within patients. The transient, low-abundance nature of trogocytosed molecules and the challenge of distinguishing them from exosome-mediated transfer or passive uptake remain significant barriers. Advanced instrumentation including high-parameter flow and mass cytometry, engineered biosensor systems and standardized liquid biopsy platforms offer potential solutions. Importantly, clinical validation should incorporate prospective trials to test the feasibility, sensitivity and reproducibility of such assays across cancer types. Thus, addressing these interconnected questions will not only deepen our biological understanding of trogocytosis but also accelerate its translation into safe, effective and patient-centered strategies within precision oncology.

## 8 Future perspective

The dual role of trogocytosis in modulating immune-tumor interactions and in serving as a conduit for intercellular communication positions it as a highly attractive target for translational exploitation. Therapeutically, its specificity and spatial precision can be harnessed for delivering targeted molecules, while diagnostically it offers a novel source of biomarkers reflective of active immune engagement within the tumor milieu. Future research will be needed to address several challenges to fully translate these concepts into the clinic. These include optimizing the engineering of effector cells for efficient and selective trogocytosis, improving detection methods for low-abundance trogocytosed markers and validating these biomarkers across diverse patient populations and cancer types.

However, significant challenges still remain. Efforts must focus on elucidating the molecular determinants that govern trogocytic specificity, stability and persistence of transferred molecules. In parallel, scalable and clinically compliant methods for detecting trogocytosed markers particularly in circulating immune-cells and extracellular vesicles need to be standardized. Importantly, the immunological consequences of trogocytosis, such as potential modulation of immune-cell function or induction of tolerance, must be carefully considered in therapeutic contexts. Looking forward, combining trogocytosis-based technologies with synthetic biology, nanomedicine and systems immunology could unlock powerful new modalities in cancer treatment and immune diagnostics. Nevertheless, trogocytosis is emerging as a pivotal element in the next-generation of cell-based therapies and immune diagnostics, driving advancements in precision oncology and personalized medicine.

## Glossary

ADCC: Antibody-dependent cellular cytotoxicity
ANO6: Anoctamin 6
APC: Antigen presenting cell
ATF: Activating Transcription Factor 3
ADCT: Antibody-dependent cellular trogocytosis
BC: Breast cancer
BCRP: Breast cancer resistant protein
CAFs: Cancer associated fibroblasts
CSC: Cancer stem cell
CAR: Chimeric Antigen Receptor
CD: Cluster of differentiation
CH25H: Cholesterol-25-Hydroxylase
CRISPR: Clustered regularly interspaced short palindromic repeats
CTLA4: Cytotoxic T-Lymphocyte-Associated Protein 4
DC: Dendritic cell dNK: Decidual like NK
EMT: Epithelial to mesenchymal transition
ECM: Extracellular matrix
Fc: Fragment crystallizable receptor
FGF-2: Fibroblast growth factor 2
HER2: Human epidermal growth factor receptor 2
HGSC: High-grade serous carcinoma
HL: Hodgkin lymphoma
HRS: Hodgkin and Reed Sternberg
ICAM: Intercellular Adhesion Molecule
IFN-γ: Interferon γ
KRAS: Kirsten rat sarcoma viral oncogene homolog
LAG3: Lymphocyte-activation gene 3
LCK: Lymphocyte specific protein tyrosine kinase
LMP-1: Latent membrane 1 protein
LRP: Lung resistant protein
MAC-1: Macrophage-1 antigen
MDR: Multi-drug resistant
MHC: Major histocompatibility complex
MMP-9: Matrix metalloproteinase 9
MRP: Multi-drug resistant protein
MSC: Mesenchymal stem cell
N-cadherin: Neural cadherin
NK cells: Natural killer cells
NKG2D: Natural Killer group 2, member D
NKp46: Natural cytotoxicity receptor 1
PBMCs: Peripheral blood mononuclear cells
PCR: Pathological complete response
PD-1: Programmed cell death protein 1
PDGF: Platelet derived growth factor
PD-L1: Programmed Death-Ligand 1
Pgp: P-glycoprotein
PS: Phosphotidyl serine
PST: Primary systemic therapy
PDAC: Pancreatic ductal adenocarcinoma
RCC: Renal cell carcinoma
SDF1: Stromal cell derived factor-1
siRNA: Small interfering RNA
SIRPα: Signal regulatory protein alpha
SMAD: Suppressor of Mothers against Decapentaplegic
TCR: T-cell receptor
TGF: Transforming growth factor
Th1: T helper 1 cell
Tregs: Regulatory T cells
TILs: Tumor-infiltrating lymphocytes
Tim3: T cell immunoglobulin and mucin domain-3
TME: Tumor-microenvironment
TNBC: Triple negative breast cancer
TLR9: Toll like receptor 9
TYRO3: Tyrosine-protein kinase receptor
VEGF: Vascular endothelial growth factor
